# Targeting PAK1 or PAK4 Uncovers Different Mechanisms of Vascular Reprogramming in Pancreatic Cancer

**DOI:** 10.3390/cells14221806

**Published:** 2025-11-17

**Authors:** Arian Ansardamavandi, Chelsea Dumesny, Sarah Ellis, Ching-Seng Ang, Mehrdad Nikfarjam, Hong He

**Affiliations:** 1 Department of Surgery, Austin Precinct, The University of Melbourne, 145 Studley Rd, Melbourne, VIC 3084, Australia; arian.ansardamavandi@student.unimelb.edu.au (A.A.); watsoncj@unimelb.edu.au (C.D.); m.nikfarjam@unimelb.edu.au (M.N.); 2 Olivia Newton-John Cancer Research Institute, Melbourne, VIC 3086, Australia; sarah.ellis@onjcri.org.au; 3 School of Cancer Medicine, La Trobe University, Melbourne, VIC 3000, Australia; 4 Mass Spectrometry and Proteomics Facility, Bio21 Molecular Science and Biotechnology Institute, University of Melbourne, Parkville, VIC 3010, Australia; ching-seng.ang@unimelb.edu.au; 5 Department of Hepatopancreatic-Biliary Surgery, Austin Health, 145 Studley Rd, Melbourne, VIC 3084, Australia

**Keywords:** pancreatic ductal adenocarcinoma, PAK1 knockdown, PAK4 knockout, tumour vasculature, hypoxia, endothelial adhesion molecules, vascular mimicry

## Abstract

The tumour microenvironment in pancreatic ductal adenocarcinoma (PDA) regulates vascular function and therapeutic response. P21-activated kinases (PAKs) regulate cytoskeletal dynamics and angiogenesis; however, their roles in vascular reprogramming and chemotherapy responses remain unclear. This study examined the effects of a PAK1 knockdown (PAK1KD) and a PAK4 knockout (PAK4KO) on vascular remodelling in PDA. Human PANC-1 wild-type (WT), PAK1KD, and PAK4KO cells were injected subcutaneously into the flanks of SCID mice followed gemcitabine treatment. The tumour growth, vascular density, pericyte coverage, adhesion molecules, and hypoxia were determined. A proteomics study was used to identify the molecular changes involved in the vascular pathways. PAK1KD suppressed tumour growth and angiogenesis, promoted vascular normalisation, reduced hypoxia, and increased stromal ICAM-1. PAK4KO inhibited tumour growth, enlarged vessels, enhanced angiogenesis, and reduced hypoxia. PAK4KO did not affect adhesion molecules in the absence of gemcitabine, but markedly upregulated ICAM-1 and VCAM-1 with gemcitabine. Additionally, PAK4KO promoted vascular mimicry (VM) with a compromised integrity in tumour-derived vessels, but enhanced the integrity in endothelial-derived vessels. The proteomics study confirmed the enrichment of molecules in fibronectin and the VEGF pathway in PAK4KO cancer cells, along with the upregulation of EphA2, RhoA, ROCK1, ROCK2, and components of the EPH-ephrin signalling pathway, linking to enhanced VM. Neither PAK1KD nor PAK4KO increased the gemcitabine efficacy. In conclusion, PAK1KD and PAK4KO suppressed tumour growth with distinct vascular effects, but failed to enhance the gemcitabine responses, suggesting that PAK targeting reprograms the PDA vasculature, but offers limited benefit in chemotherapy-resistant models.

## 1. Introduction

Pancreatic ductal adenocarcinoma (PDA) is a deadly malignancy with a very low 5-year survival rate due to limited diagnostic methods and a lack of effective therapies [1]. Effective new therapy development is urgently needed for pancreatic cancer, as the current options such as FOLFIRINOX and gemcitabine-based regimens provide only limited benefit and often cause severe side effects [2]. The limited efficacy of gemcitabine in PDA is affected by microenvironment-mediated resistance mechanisms [3].

PDA is characterised by an excessively dense extracellular matrix, vasculature collapse, an impaired microvessel integrity, and poorly perfused vessels, contributing to its resistance to therapies [4,5]. Conventional anti-angiogenic therapies have shown limited efficacy in PDA, largely due to the abnormal vasculature in the tumour microenvironment (TME) [4]. Pancreatic cancer overexpresses proangiogenic proteins such as vascular endothelial growth factor A (VEGFA), contributing to treatment resistance.

PDA is mainly driven by KRAS mutations, and new therapies targeting KRAS and its signalling are being developed [6]. P21-activated kinases (PAKs) are important downstream effectors of KRAS in pancreatic cancer [7]. PAKs consist of two families, PAKs 1–3 and PAKs 4–6, and they play important roles in cytoskeletal organisation and cell growth [8]. PAK1 and/or PAK4 are overexpressed in pancreatic cancer and play a crucial role in its progression, becoming a potential therapeutic target [9,10]. PAK1 has been implicated in cytoskeletal remodelling and endothelial barrier regulation, whereas PAK4 is linked to cancer cell survival, stemness, and therapy resistance [11,12]. Targeting PAK has inhibited cancer cell growth, improved chemotherapy efficacy, and enhanced anti-tumour immunity through blood vessel normalisation [13]. For instance, targeting PAK4 normalises the tumour vascular microenvironment and improves CAR-T immunotherapy for glioblastoma [14]. The inhibition of PAK1 or PAK4 suppresses pancreatic cancer by stimulating anti-tumour immunity through the activation of T cells and dendritic cells through ICAM-1 and VCAM-1 upregulations [15]. ICAM-1 and VCAM-1 are key adhesion molecules that mediate leukocyte adhesion and migration across the endothelium, thereby regulating the immune responses, inflammation, and tumour progression [16].

PAK signalling influences endothelial behaviour and immune functions [13]; however, the specific roles of PAK1 or PAK4 in vascular reprogramming, hypoxia, and the gemcitabine response have not been fully addressed in pancreatic cancer. An immunodeficient xenografted tumour model, where adaptive immunity is absent and immune–vascular crosstalk is restricted, allows an assessment of the direct interaction between the tumour and the endothelium [17,18]. Elucidating these mechanisms may support the development of strategies aimed at vascular normalisation and improved drug delivery. We used PANC-1 human pancreatic cancer cell tumour xenografts in SCID mice to assess the effect of PAK modification on the tumour vasculature and the drug response in the absence of adaptive immunity. PANC-1 was selected because it represents a gemcitabine-resistant phenotype, providing a model to evaluate the effect of PAK modification on the gemcitabine responses in a chemoresistant tumour type [19,20].

## 2. Materials and Methods

### 2.1. Cell Lines and Cell Culture

PAK1KD PANC-1 cells were generated by transfection with SureSilencing shRNA plasmids targeting human PAK1 (SABiosciences, Melbourne, VIC, Australia) or with a scrambled sequence as a negative control (NC), using Lipofectamine 2000 (Invitrogen, Melbourne, VIC, Australia) according to the manufacturer’s instructions. Stable clones were selected in geneticin (G418; 1 mg/mL), and the knockdown efficiency was validated by a Western blot, as described in our previous publication [21].

For PAK4KO, PANC-1 cells were generated using an inducible lentiviral CRISPR-Cas9 system. Briefly, the pFgH1tUTG-GFP lentiviral vector carrying single guide RNAs (sgRNAs) targeting human PAK4 (guide 1: GCAGCCGAGGCCGGTTCGC; guide 2: GCTTCGACCAGCACGAGCAG) was transfected into PANC-1 cells, and single-cell clones were isolated by fluorescence-activated cell sorting (FACS) using a BD FACS Aria III (BD Biosciences, Jersey City, NJ, USA). PAK4KO was verified by immunoblotting, and CRISPR editing events were confirmed, as reported in our previous publication [22]. All the cell lines were maintained in Dulbecco’s modified Eagle’s medium (DMEM) supplemented with 5% foetal bovine serum (FBS) (Thermo Fisher Scientific, Melbourne, Australia) and kept at 37 °C in a humidified atmosphere containing 5% CO_2_.

### 2.2. Animal Studies

All the animal experiments were approved by the Austin Health Animal Ethics Committee (approval numbers A2022-05797 and A2023-05849) and carried out in accordance with institutional guidelines. Male SCID mice (7 weeks old) were housed in the Austin Health Bioresource Facility under standard conditions with routine health monitoring.

PANC-1 WT, PANC-1 PAK1KD, or PANC-1 PAK4KO cells (0.5–1 × 10^6^ cells suspended in 100 μL/mouse) were injected subcutaneously into both flanks. Eight mice were used in the WT, PAK1KD, and PAK4KO groups, respectively. In each group, the mice were randomly assigned to the gemcitabine treatment group (n = 4) or the control group (n = 4). The treatment cohort received gemcitabine intraperitoneally at 50 mg/kg twice weekly for up to 6 weeks, while the control group was injected with saline. The tumour volumes were monitored with digital callipers, and the volumes were calculated using the following formula: volume (V) = length (L) × width (W)^2^ × 0.5. At the study endpoint, the animals were euthanised, the tumours were excised, and the tumour weights (g) were recorded.

To measure the tumour-associated vessel diameter, the blood vessels adjacent to each tumour were exposed at the time of collection and photographed with a ruler for calibration. The diameters were quantified in ImageJ (Java v1.8.0_322) by drawing a straight line across the inner edges of each vessel’s cross-section. Measurements were performed by an independent observer blinded to the group allocation, and the mean diameter of multiple vessels per tumour was used for the analysis.

### 2.3. Immunohistochemistry

Tumour tissues were fixed in formalin, embedded in paraffin, and sectioned at a 5 μm thickness using a LEICA RM2245 microtome (Leica Biosystems, Nussloch, Germany). Heat-induced antigen retrieval was performed by immersing the slides in 10 mM Tris-EDTA buffer (pH of 9.0) at 99 °C for 30 min, followed by cooling to room temperature for 30 min (Table S1). Endogenous peroxidase activity was quenched with Dako REAL^TM^ peroxidase blocking solution (S2023, Agilent Technologies, Glostrup, Denmark) for 15 min in the dark. To minimise non-specific antibody binding, the sections were incubated for 1 h at room temperature with a blocking buffer containing 5% normal goat serum (NGS) and 1% bovine serum albumin (BSA) in TBS-T (Table S1).

The slides were then incubated overnight at 4 °C with the relevant primary antibodies (Table S2), followed the next day by exposure to an HRP-conjugated goat anti-rabbit polymer (K4003, EnVision+ System-HRP (DAB) kit, K4003, Dako, Agilent Technologies) for 1 h. Antibody binding was visualised using the EnVision FLEX DAB+ Substrate Chromogen System (K3468, Dako, Agilent Technologies), and the nuclei were counterstained with haematoxylin (S3309, Dako, Agilent Technologies). After rehydration, the slides were mounted with DPX medium (06522, Sigma-Aldrich, St. Louis, MO, USA) and left to dry for 24 h before imaging.

Whole-slide brightfield images were acquired on the Aperio AT2 scanner (Leica Biosystems, Nussloch, Germany). A quantitative image analysis was conducted using the HALO software, v4.1 (Indica Labs). A fixed-intensity threshold was applied across all samples to ensure consistency.

The microvessel density (MVD) of large vessels was evaluated on haematoxylin and eosin (H&E)-stained tumour sections. All the slides were scanned at 20× magnification (0.5 µm/pixel) using the Aperio AT2 digital slide scanner (Leica Biosystems). The entire scanned tumour area was analysed. Clusters of red blood cells (RBCs), indicative of vascular lumina, were manually identified by an independent observer blinded to group allocation. Vessels containing more than five RBCs were classified as large vessels. The MVD of large vessels (%) was calculated by dividing the total number of large vessels by the total stained tissue area. This manual approach ensured the accurate identification of perfused large vessels and the exclusion of necrotic or haemorrhagic regions that could interfere with automated image-based quantification.

For the vascular mimicry (VM) assessment, after IHC staining for CD31, the sections were washed with running water and stained using a periodic acid–Schiff (PAS) staining kit (ab150680, Abcam, Cambridge, UK). CD31 staining was performed to identify endothelial cells, and any structure containing CD31-positive immunoreactivity was defined as a blood vessel. PAS staining was used to identify matrix-associated vascular channels in the pancreatic cancer tissues. VM structures were defined as CD31-negative, PAS-positive structures using the HALO software, v4.1 (Indica Labs) [23].

### 2.4. Immunofluorescence

Paraffin-embedded tumour sections underwent antigen retrieval and blocking before overnight incubation at 4 °C with anti-CD31 antibody. After washing, the slides were incubated with an HRP-conjugated goat anti-rabbit secondary antibody for 1 h, followed by tyramide signal amplification using a fluorophore at either Alexa Fluor^TM^ 488 (B40922; Thermo Fisher Scientific, Waltham, MA, USA) or Alexa Fluor^TM^ 647 (B40926; Thermo Fisher Scientific) for 10 min.

The slides were then subjected to a second round of antigen retrieval and blocking, followed by overnight incubation with anti-NG2 or anti-α-SMA antibodies. The bound antibodies were detected using HRP-conjugated secondary antibodies and labelled with a spectrally distinct fluorophore. For sequential labelling, the fluorophores were changed to prevent spectral overlap (Alexa Fluor^TM^ 594 (B40925; Thermo Fisher Scientific) when Alexa Fluor^TM^ 488 was used first, or Alexa Fluor^TM^ 488 when Alexa Fluor^TM^ 647 was applied first).

For multiplex staining to assess the vascular integrity, the sections were stained sequentially for CD31 (endothelial marker), VE-cadherin, and cytokeratin 19 (tumour cell marker) using compatible fluorophores (Alexa Fluor^TM^ 647, 555 (B40923; Thermo Fisher Scientific), and 488) (Table S4). Nuclear counterstaining was performed with DAPI (FP1490, Akoya Biosciences, Marlborough, MA, USA), and the slides were mounted with VECTASHIELD antifade medium (H-1700, Vector Laboratories, Burlingame, CA, USA). Whole-slide fluorescence images were acquired using the Zeiss Axioscan 7 slide scanner (Carl Zeiss AG, Oberkochen, Germany). A quantitative analysis was carried out with the HALO area quantification FL module, v3.0.1 (Indica Labs, Albuquerque, NM, USA), and the thresholds were adjusted individually to account for variability between samples and ensure accurate quantification.

### 2.5. Western Blot

PANC-1 WT, PANC-1 PAK1KD, and PANC-1 PAK4KO cells were seeded into 24-well plates and cultured for 48 h before lysis. The cells were lysed directly in 2× loading buffer (Table S1), and equal amounts of protein extracts were separated on 10% SDS–PAGE gels followed by transfer to nitrocellulose membranes.

The membranes were blocked and probed with primary antibodies against ICAM-1, VCAM-1, VEGFA, VE-cadherin, PAK1, PAK4, and GAPDH. After washing, the membranes were incubated with an HRP-conjugated goat anti-rabbit IgG secondary antibody (1706515, Bio-Rad, Hercules, CA, USA). The protein bands were visualised using the ECL Select^TM^ chemiluminescent substrate (RPN2235, Cytiva, Amersham, UK) and imaged with the ChemiDoc^TM^ MP Imaging System (Bio-Rad Laboratories, Hercules, CA, USA). The band intensities were quantified in ImageJ (Java 1.8.0_322), and the protein expression levels were normalised to GAPDH as a loading control.

### 2.6. Tube Formation Assay

PANC-1 WT, PANC-1 PAK1KD, and PANC-1 PAK4KO cells were seeded into 24-well plates at 5 × 10^4^ cells/well in 1.2 mL of DMEM supplemented with 5% FBS and cultured for 24 h. The medium was then replaced with serum-free DMEM for another 24 h. Conditioned medium (CM) was harvested and centrifuged at 400× *g* for 5 min to remove debris, and the clarified supernatant was used immediately.

Geltrex^TM^ Basement Membrane Matrix (A1413202, Thermo Fisher Scientific) was thawed at 4 °C overnight and handled on ice. A pre-chilled 24-well plate was pre-coated with 0.1 mL Geltrex and incubated at 37 °C for 30 min to allow polymerisation. Human umbilical vein endothelial cells (HUVECs) were washed with PBS, detached using TrypLE^TM^ Express (12604013; Gibco, Thermo Fisher Scientific), and resuspended in CM at a density of 2 × 10^5^ cells/mL. Approximately 0.25 mL of suspension (~5 × 10^4^ cells) was seeded onto each coated well.

Tube formation was evaluated after 14–20 h of incubation at 37 °C in 5% CO_2_. Network structures were visualised using an inverted microscope at 4× magnification, and the number of tubes was quantified manually by an observer blinded to the treatment groups. Each treatment was tested in triplicate, and the results were compared to determine the effects of the CM from different PANC-1 lines on the endothelial tube-forming capacity.

### 2.7. Proteomics

The proteomic analysis included sample preparation, liquid chromatography–mass spectrometry with data-independent acquisition (LC–DIA-MS), database searching, and a statistical analysis to identify differentially expressed proteins. For sample preparation, PANC-1 WT, PANC-1 PAK1KD, PANC-1 PAK4KO, and NC PANC-1 cells were seeded into 10 cm culture dishes and cultured to ~80% confluence. The cells were lysed in RIPA buffer supplemented with protease and phosphatase inhibitors (Roche, Mannheim, Germany). Proteins were precipitated with acetone, enzymatically digested into peptides, and analysed with LC–DIA-MS by following established protocols [24].

The raw DIA MS data were searched using Spectronaut (version 19.9.250324) on the Uniprot *Homo sapiens* reference database, followed by a statistical analysis using the Perseus software (v2.1.3.0). The label-free quantification (LFQ) intensity values were log_2_-transformed, and the proteins were retained for analysis only when at least three valid values per group (n = 4) were available. Differentially abundant proteins were identified using a two-sample *t*-test with a background variance parameter S_0_ = 0.1 and a false discovery rate (FDR) threshold of <0.05 [25]. Volcano plots were generated under these parameters, displaying –log_10_ *p*-values (y-axis) against log_2_ fold changes (x-axis), with significantly altered proteins highlighted.

Protein–protein interaction (PPI) networks were constructed in Cytoscape (v3.10.3) using the STRING app (v2.2.0) with the *Homo sapiens* database and a confidence score cut-off of 0.7. Functional enrichment and a pathway analysis were performed across multiple categories, and selected pathways of interest are presented. In the visualisations, upregulated proteins are shown in red and downregulated proteins in blue using a continuous colour scale.

### 2.8. Statistical Analysis

Quantitative data are presented as the mean ± standard error of the mean (SEM). All data were tested for consistency across the experimental groups, with staining, imaging, and quantification performed under identical conditions to minimise variability. Because the datasets exhibited a comparable variance and approximately normal distributions, parametric statistical tests (*t*-test or one-way ANOVA) were applied. These tests are considered robust to minor deviations from normality when the experimental conditions are uniform and the sample sizes are balanced [26,27]. In this regard, comparisons between the two groups were assessed using unpaired, two-tailed Student’s *t*-tests, assuming a normal distribution and equal variance. For experiments involving three or more groups, a one-way ANOVA was applied, with pairwise comparisons performed using Fisher’s least significant difference (LSD) test. When two independent variables were evaluated simultaneously, a two-way ANOVA with an interaction term was used, followed by Fisher’s LSD post hoc testing with a single pooled variance. A 95% confidence level was applied, and statistical significance was considered at *p* < 0.05. All the analyses were conducted using the GraphPad Prism software, version 10.5.0 (GraphPad Software, San Diego, CA, USA).

## 3. Results

### 3.1. PAK1KD Reduced Tumour Growth and Angiogenesis, but Did Not Increase the Inhibitory Effect of Gemcitabine

**Figure 1:**
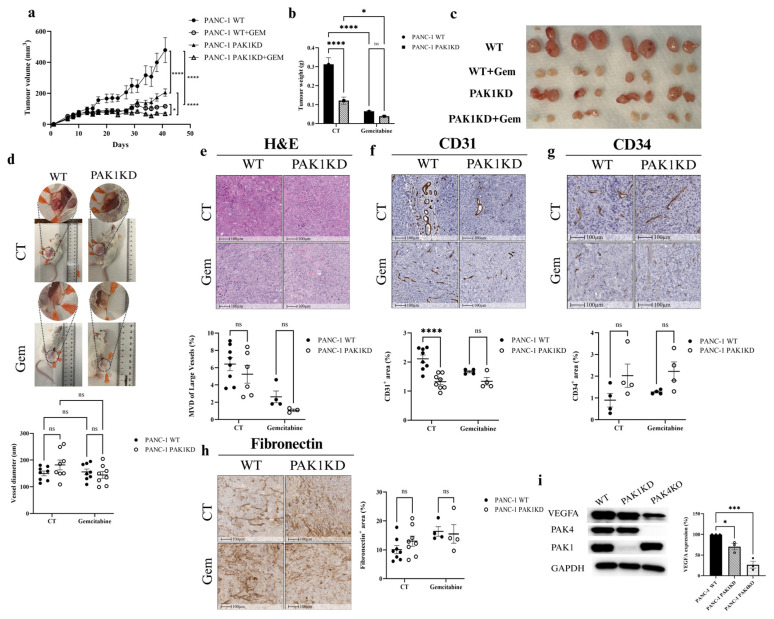
PAK1KD reduced tumour growth and angiogenesis in PANC-1-xenografted tumours. PANC-1 WT and PAK1KD cells were injected subcutaneously into SCID mice (8 mice per group) and treated ± gemcitabine (50 mg/kg, twice weekly). (**a**–**c**) Tumour volume and weight were significantly reduced by PAK1KD or gemcitabine. (**d**,**e**) Vessel diameter and microvessel density of large vessels remained unchanged. (**f**,**g**) Angiogenesis was reduced in PAK1KD tumours, as indicated by decreased CD31 expression, while CD34 levels remained unchanged. (**h**) Fibronectin levels were unchanged. (**i**) VEGFA expression was decreased in PAK1KD cancer cells, suggesting a mechanism underlying the reduced angiogenesis in this group. WT: wild type; KD: knockdown; KO: knockout; CT: control. * *p* < 0.05, *** *p* < 0.001, **** *p* < 0.0001; ns: not significant.

PAK1KD suppressed pancreatic tumour growth in the SCID mouse model, as shown by reductions in the tumour volume and weight (Figure 1a–c). PAK1KD also suppressed tumour angiogenesis by reducing the expression of the endothelial marker CD31 (Figure 1f), not CD34 (Figure 1g). Both CD31 and CD34 were used as endothelial markers to assess angiogenesis, since they show heterogeneous, but complementary, expression across mature and immature vascular beds. Considering both provided a more reliable and comprehensive assessment of angiogenesis in PDA tumours [28,29,30,31]. Fibronectin deposition remained unchanged between the PAK1KD and WT tumours (Figure 1h). PAK1KD did not change the vessel diameter or the MVD of the large vessel with or without gemcitabine (Figure 1d,e). Moreover, the VEGFA expression, a key pro-angiogenic factor in cancer cells [32], was markedly reduced in PAK1KD cells (Figure 1i), suggesting a reduced angiogenic potential following PAK1 suppression.

Gemcitabine inhibited the growth of both WT and PAK1KO tumours (Figure 1a–c), but did not affect the expression of CD31 or CD34 (Figure 1f,g). PAK1KD did not change the inhibitory effects of gemcitabine (Figure 1b). The results suggested that PAK1KD inhibited angiogenesis and tumour growth without affecting the gemcitabine effect in the immunocompromised (SCID) model. Our previous report demonstrated that using FRAX597 (PAK1 inhibitor) with gemcitabine produced the maximal inhibitory effect on tumour growth in an immunocompetent (C57BL/6) murine orthotopic pancreatic cancer model [21]. Together, these findings highlight the critical role of the immune system in shaping tumour responses to chemotherapy.

### 3.2. PAK1KD Increased ICAM-1 Expression and Promoted Vascular Normalisation, Followed by Reduced Hypoxia

**Figure 2:**
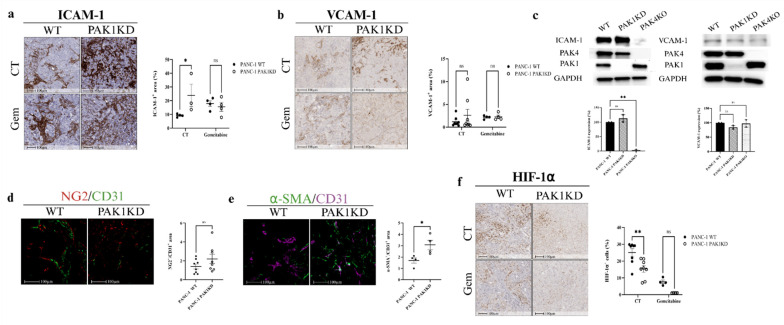
Dupregulated ICAM-1 and enhanced vascular normalisation in PANC-1-xenografted tumours, and reduced hypoxia. Immunohistochemistry of tumour tissues showed increased ICAM-1 (**a**), but no change in VCAM-1 (**b**) within PAK1KD compared to WT tumours. (**c**) ICAM-1 and VCAM-1 expression did not change in PAK1KD cancer cells, while PAK4KO reduced ICAM-1 expression, indicating that increased ICAM-1 was primarily driven by stroma cells in PAK1KD tumours. (**d**,**e**) Vascular normalisation was assessed by NG2/CD31 and α-SMA/CD31 ratios; PAK1KD significantly increased α-SMA/CD31, suggesting greater pericyte coverage. (**f**) Increased vascular normalisation, followed by reduced hypoxia, e.g., HIF-1a expression in the PAK1KD tumours. WT: wild type; KD: knockdown; KO: knockout; CT: control. * *p* < 0.05, ** *p* < 0.01; ns: not significant.

Endothelial adhesion molecules regulate endothelial permeability, maturation, and lymphocyte trafficking [33,34]. PAK1KD increased the ICAM-1 expression, but not VCAM-1 (Figure 2a,b), suggesting a targeted activation of ICAM-1-mediated endothelial–leukocyte interactions without a VCAM-1 response. Although tumour cells are also capable of expressing ICAM-1 and VCAM-1, PAK1KD did not change the ICAM-1 or VCAM-1 expressions in pancreatic cancer cells (Figure 2c). These findings suggest that the upregulation of ICAM-1 observed in PAK1KD tumours is primarily driven by stromal, rather than tumour, compartments.

Vascular normalisation is typically defined by enhanced pericyte coverage, which is reflected by an increased ratio of mature pericyte markers such as NG2 or α-SMA to the endothelial marker CD31 [35]. In pancreatic tumour tissues, PAK1KD significantly elevated pericyte coverage, as demonstrated by a higher α-SMA/CD31 ratio (Figure 2e), whereas the NG2/CD31 ratio remained unchanged (Figure 2d). Following vascular normalisation, the hypoxia level measured by the HIF-1α marker was reduced significantly in PAK1KD tumours (Figure 2f). Collectively, these findings suggest that PAK1KD led to stromal ICAM-1 expression and vascular normalisation, associated with reduced hypoxia in the TME.

### 3.3. PAK4KO Reduced Tumour Growth and Increased Angiogenesis, but Did Not Increase the Inhibitory Effect of Gemcitabine

**Figure 3:**
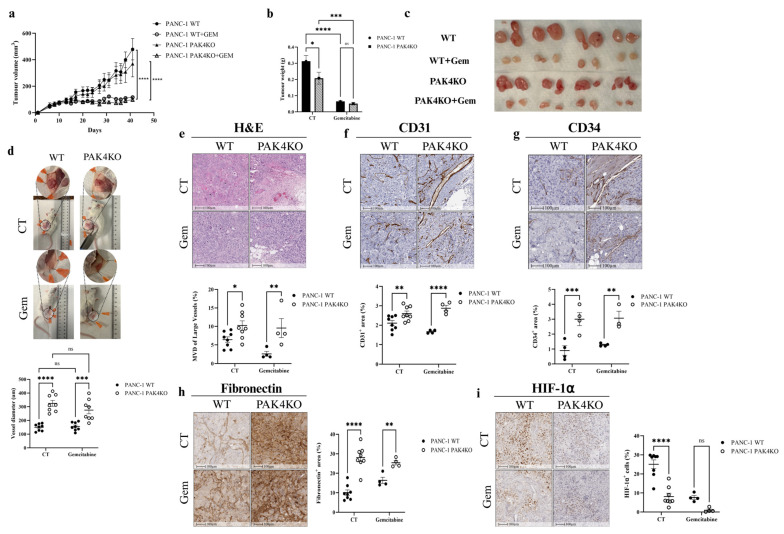
PAK4KO reduces tumour growth and increases angiogenesis in PANC-1-xenografted tumours and is associated with reduced hypoxia, but did not stimulate gemcitabine efficacy. PANC-1 WT and PAK4KO cells (n = 4 mice per treatment per group) were injected subcutaneously into SCID mice and treated ± gemcitabine (50 mg/kg, twice weekly). (**a**–**c**) Tumour volume and weight were reduced by PAK4KO or gemcitabine. PAK4KO did not increase inhibitory effect of gemcitabine. (**d**,**e**) Vessel diameter and microvessel density (MVD) of large vessels increased in PAK4KO tumours. (**f**,**g**) Angiogenesis increased in PAK4KO tumours, as measured by CD31 and CD34. (**h**) Increased angiogenesis was associated with higher fibronectin deposition in PAK4KO tumours. (**i**) Increased angiogenesis was also associated with reduced hypoxia (HIF-1α) in PAK4KO tumours. WT: wild type; KO: knockout; CT: control. * *p* < 0.05, ** *p* < 0.01, *** *p* < 0.001, **** *p* < 0.0001; ns: not significant.

PAK4KO reduced the tumour weight (Figure 3b,c), but not the tumour volume (Figure 3a). PAK4KO enlarged the vessel diameter surrounding the tumours, as quantified using ImageJ (Figure 3d). The MVD of large vessels was also increased significantly in PAK4KO tumours (Figure 3e). This was accompanied by increased angiogenesis, as reflected by elevated CD31 and CD34 expression (Figure 3f,g), with higher fibronectin deposition in PAK4KO tumours (Figure 3h). The increased angiogenesis in PAK4KO tumours was associated with a reduction in hypoxia, as shown by a decreased HIF-1α expression (Figure 3i). Interestingly, despite this higher angiogenesis in PAK4KO tumours, the VEGFA expression in PAK4KO tumour cells was reduced compared to the WT, suggesting the primary role of fibronectin in promoting angiogenesis by the PAK4 knockout (Figure 1h,i).

Gemcitabine suppressed pancreatic tumour growth in both WT and PAK4KO tumours, as evidenced by a decreased tumour size and mass (Figure 3a–c). The gemcitabine efficacy was not changed in PAK4KO tumours (Figure 3b). In the gemcitabine-treated group, PAK4KO increased the vessel diameter, the MVD of large vessels, angiogenesis (CD31 and CD34 expression), and the fibronectin levels, showing a trend similar to the control group (without gemcitabine) (Figure 3d–h). It also reduced hypoxia, but did not reach a significant change, probably because of the limited sample size (Figure 3i). Collectively, these results suggest that PAK4KO decreased the tumour weight, increased the vessel diameter and angiogenesis with higher fibronectin deposition, and reduced hypoxia, but did not affect the gemcitabine efficacy.

### 3.4. PAK4KO Increased ICAM-1 and VCAM-1 Expression in the Presence of Gemcitabine, but Did Not Promote Vascular Normalisation

**Figure 4:**
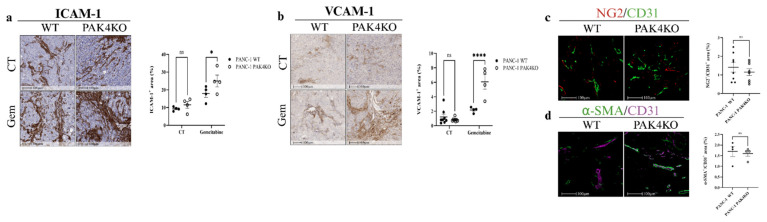
PAK4KO did not affect vascular normalisation, but increased endothelial adhesion molecules in the gemcitabine-treated group. In control tumours, ICAM-1 (**a**) and VCAM-1 (**b**) were unchanged, but both were significantly increased in PAK4KO tumours following gemcitabine treatment. (**c**,**d**) The vascular normalisation markers and the NG2/CD31 and α-SMA/CD31 ratios were unaffected by PAK4KO. WT: wild type; KO: knockout; CT: control. * *p* < 0.05, **** *p* < 0.0001; ns: not significant.

To further assess the immune permissive TME by PAK4KO, we assessed the ICAM-1 and VCAM-1 expression. In pancreatic tumour tissues, PAK4KO did not change the ICAM-1 (Figure 4a) or VCAM-1 (Figure 4b) expression in the absence of gemcitabine, nor did it affect vascular normalisation, as shown by the unchanged NG2/CD31 (Figure 4c) and α-SMA/CD31 (Figure 4d) ratios. However, under gemcitabine treatment, PAK4KO significantly upregulated both ICAM-1 and VCAM-1 compared with WT tumours (Figure 4a,b), suggesting an activation of these molecules following the gemcitabine treatment. Interestingly, PAK4KO reduced the ICAM-1 expression in pancreatic cancer cells, while the VCAM-1 expression was unaffected compared to the WT (Figure 2c), showing that the upregulated ICAM-1 and VCAM-1 in the gemcitabine-treated PAK4KO tumours were expressed in stroma cells rather than cancer cells. Together, these findings suggest that PAK4KO did not intrinsically regulate endothelial adhesion molecules or vascular normalisation within tumour tissues. However, in the context of gemcitabine treatment, PAK4KO led to stromal or vascular changes associated with ICAM-1 and VCAM-1 upregulation.

**Figure 5:**
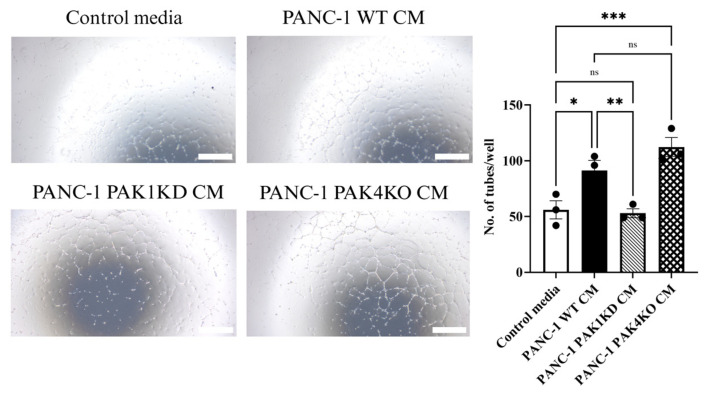
Conditioned media (CM) from PAK1KD decreased endothelial tube formation. HUVECs were cultured in DMEM or CM from PANC-1 WT, PAK1KD, or PAK4KO cells. PAK1KD CM significantly reduced tube formation compared to the WT, whereas WT and PAK4KO CM increased the tube-forming capacity compared to the control media. WT: wild type; KD: knockdown; KO: knockout; CM: conditioned media. * *p* < 0.05, ** *p* < 0.01, *** *p* < 0.001; ns: not significant. Scale bar: 1000 µm.

The impact of PAK modulation on endothelial behaviour was further investigated by culturing human endothelial cells (HUVECs) with conditioned medium (CM) derived from pancreatic cancer cells, followed by an assessment of their tube-forming capacity. CM from WT and PAK4KO cells markedly promoted endothelial tube formation compared to the control, whereas CM from PAK1KD cells significantly reduced tube formation compared with WT CM (Figure 5).

### 3.5. PAK4 Knockout Promoted Vascular Mimicry with Compromised Integrity in Tumour-Derived Vessels, but Enhanced Integrity in Endothelial-Derived Vessels

Vascular Mimicry (VM) is a process whereby tumour cells form de novo vessel-like structures to facilitate blood supply and nutrient delivery within the tumour, independent of traditional endothelial cell-lined angiogenesis [23,36]. This mechanism is particularly prevalent in aggressive malignancies like PDA, where it contributes to tumour progression and therapy resistance by providing alternative perfusion pathways [37,38].

**Figure 6:**
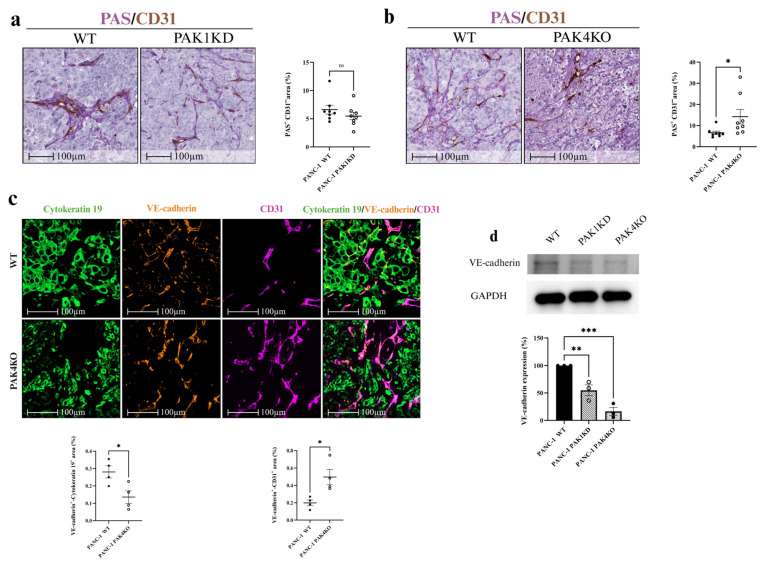
PAK4KO promoted vascular mimicry with compromised integrity in tumour-derived vessels, but enhanced integrity in endothelial-derived vessels. (**a**,**b**) VM quantification using CD31/PAS staining in PAK1KD and PAK4KO tumours. (**c**) Multiplex immunofluorescence showing VE-cadherin expression in cytokeratin 19^+^ (tumour) and CD31^+^ (endothelial) areas. (**d**) Western blot of VE-cadherin in cancer cells. VM: vascular mimicry; WT: wild type; KD: knockdown; KO: knockout. * *p* < 0.05, ** *p* < 0.01, *** *p* < 0.001; ns: not significant.

To determine whether VM structures were present, we utilised anti-CD31 and PAS staining to identify endothelium and VM channels, respectively. CD31-positive staining marked vessels formed by endothelial cells, whereas CD31-negative, PAS-positive vascular-like structures containing red blood cells, formed by cancer cells or the extracellular matrix (ECM), were classified as VM. PAK1KD did not affect VM-formed vessels (Figure 6a), whereas PAK4KO significantly upregulated VM structures (Figure 6b). Thus, PAK4KO not only promoted angiogenesis, but also enhanced vascular structures formed by cancer cells (Figure 3 and Figure 6), potentially compensating for alterations in traditional angiogenic pathways and contributing to sustained tumour perfusion.

To further evaluate the vascular integrity in PAK4KO tumours, we employed multiplex immunofluorescence to assess the VE-cadherin expression as a marker of the vascular integrity in tumour tissues [39] We used cytokeratin 19 as a tumour cell marker and CD31 as an endothelial cell marker. VM structures formed by cancer cells exhibited a lower vascular integrity, as shown by a decreased cytokeratin 19+/VE-cadherin+ area, while vessels formed by endothelial cells showed a significantly higher vascular integrity, as represented by an increased VE-cadherin+/CD31+ area (Figure 6c). This dichotomy suggested that PAK4KO impaired the junctional stability in tumour-derived VM channels, but enhanced it in endothelial-lined vessels, possibly through the differential regulation of adhesion molecules or compensatory stromal responses. Furthermore, PAK1KD and PAK4KO significantly reduced the VE-cadherin expression in cancer cells (Figure 6d), aligning with the compromised integrity observed in VM structures.

### 3.6. Global Proteomic Profiling Revealed Distinct Pathway Changes in PAK1KD and PAK4KO Cancer Cells

The above findings demonstrate that PAK1KD and PAK4KO both suppressed tumour growth, with vascular modifications contributing to a decrease in hypoxia in the tumour. To further elucidate the molecular mechanisms underlying these effects, we performed a global proteomic analysis comparing the protein expression profiles of PAK1KD and PAK4KO cancer cells to WT controls. Differentially expressed proteins were visualised by volcano plots (Figure 6a,c). The proteomic analysis showed that the fibronectin expression was significantly increased by about 2-fold in PAK4KO cancer cells (Figure 6c), suggesting that higher fibronectin deposition in PAK4KO tumours originates from cancer cells (Figure 3h).

A protein–protein interaction (PPI) network analysis of the PAK1KD proteome revealed significant changes in proteins related to the VEGFA–VEGFR2 signalling pathway (reactome pathways) (Figure 6b). In contrast, an analysis of the PAK4KO proteome suggested the upregulation of molecules involved in the VEGF signalling pathway (KEGG pathways), further supporting the enhanced angiogenesis noted in PAK4KO tumours (Figure 3d–g and Figure 6d).

**Figure 7:**
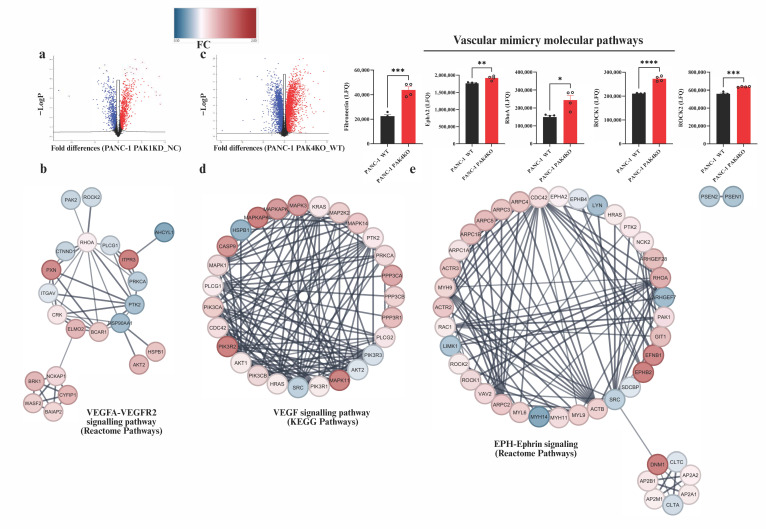
Proteomic profiling of PAK1KD and PAK4KO cells revealed different changes in signalling pathways. Global proteomics compared PANC-1 PAK1KD and PAK4KO cells with negative control or WT control. (**a**,**c**) Volcano plots show differentially expressed proteins, with red indicating upregulation and blue indicating downregulation. (**b**) PPI network analysis of PAK1KD cells identified changes in VEGFA–VEGFR2 signalling pathway (reactome). (**c**) Proteomic analysis of PAK4KO cells demonstrated an approximate two-fold increase in fibronectin expression and a significant increase in EphA2 expression, suggesting that elevated fibronectin deposition observed in PAK4KO tumours originates from cancer cells and linking EphA2 to enhanced vascular mimicry. Additionally, RhoA, ROCK1, and ROCK2 were significantly upregulated in PAK4KO cells, further supporting enhanced VM phenotype. (**d**) PPI network analysis of PAK4KO cells showed an elevation in VEGF signalling pathway molecules (KEGG) and molecules involved in EPH-ephrin signalling pathway (**e**). WT: wild type; NC: negative control; KD: knockdown; KO: knockout; FC: fold change. * *p* < 0.05, ** *p* < 0.01, *** *p* < 0.001, **** *p* < 0.0001.

The proteomic results also indicated that the ephrin type-A receptor 2 (EphA2) expression was significantly increased in PAK4KO cancer cells (Figure 7c). EphA2 expression is linked to the higher vascular mimicry noted in PAK4KO tumours. The protein–protein interactions further highlighted that the molecules involved in EPH-ephrin signalling pathways were upregulated in PAK4KO cancer cells (Figure 7e). The EPH-ephrin signalling pathway involves Eph receptor tyrosine kinases and their membrane-bound ephrin ligands, mediating bidirectional cell–cell communication essential for various developmental and pathological processes [40]. This pathway plays a primary role in angiogenesis by regulating endothelial cell migration, adhesion, vascular assembly, and remodelling [41,42]. In the context of cancer, EPH-ephrin signalling also contributes to vascular mimicry, where tumour cells form vessel-like structures to support perfusion, particularly in aggressive tumours such as glioblastoma and melanoma [43,44].

Furthermore, the proteomic analysis revealed the significant upregulation of Ras homolog family member A (RhoA) and Rho-associated coiled-coil containing kinases 1 and 2 (ROCK1 and ROCK2) in PAK4KO cancer cells (Figure 7c), providing additional molecular evidence supporting the enhanced VM observed in PAK4KO tumours. The RhoA/ROCK pathway is known to regulate cytoskeletal dynamics, cell migration, and contraction, which are critical for VM formation [45,46,47].

## 4. Discussion

Previous studies have demonstrated that PAK1 and PAK4 contribute to pancreatic cancer progression through diverse mechanisms, including the regulation of proliferation, angiogenesis, and immunity [13,15]. Both PAK1 and PAK4 belong to the p21-activated kinase family and are downstream effectors of the small GTPases Cdc42 and Rac1, which are members of the p21 family [48]. The activation of these GTPases induces conformational changes in PAKs, relieving autoinhibition and triggering kinase activation. Through this p21-dependent mechanism, PAKs regulate cytoskeletal organisation, endothelial junction stability, and angiogenic signalling [13]. The distinct vascular phenotypes observed following PAK1KD or PAK4KO may therefore reflect the differential modulation of p21 GTPase-mediated pathways, linking PAK activation to endothelial behaviour and tumour vascular remodelling.

PAK1KD suppressed tumour growth (Figure 1a–c) and decreased angiogenesis (Figure 1f). The reduction in blood vessel formation was associated with decreased VEGF expression in cancer cells, not fibronectin deposition (Figure 1h,i). Despite the reduction in vascular density, PAK1KD enhanced the vascular normalisation by increasing the α-SMA/CD31 ratio [35,49] and increased stromal ICAM-1 expression (Figure 2a,e), which is suggestive of stromal or endothelial activation in PAK1KD tumours [50]. In cancer cells, a decreased VEGFA expression directly affects blood vessel formation and maturation [51]. Anti-VEGF therapy normalises tumour vasculature, improving the delivery and efficacy of cancer treatments [52]. Likewise, PAK1KD cancer cells exhibited reduced VEGFA levels, contributing to the reduced angiogenesis and increased vascular normalisation observed in PAK1KD tumours (Figure 1f and Figure 2e). Consistently, PAK1KD CM reduced the tube-forming capacity of endothelial cells (Figure 5), further highlighting the role of PAK1 in promoting angiogenesis through cancer-cell-derived paracrine signalling. Importantly, the hypoxia levels were reduced, despite a lower vessel density (Figure 1f and Figure 2f), indicating that the remaining vasculature in PAK1KD tumours was more functionally normalised, thereby generating a less hypoxic TME that further contributed to growth suppression (Figure 2e) [53,54].

PAK4KO also reduced tumour growth (Figure 3b,c) associated with a distinct pattern of vascular remodelling compared with PAK1KD. Unlike PAK1KD tumours, PAK4KO increased angiogenesis, as evidenced by an enlarged vessel diameter, an increased MVD of large vessels, and elevated CD31 and CD34 expression (Figure 3d–g) associated with increased fibronectin deposition within the tumours (Figure 3h).

Interestingly, despite this pro-angiogenic phenotype, VEGFA expression was reduced in PAK4KO cancer cells (Figure 1i), indicating that alternative angiogenic mechanisms may compensate for VEGFA downregulation [55]. Specifically, fibronectin promotes the elongation of microvessels during angiogenesis by stimulating the adhesion-dependent migratory recruitment of endothelial cells [56], suggesting an alternative mechanism to the increased angiogenesis in PAK4KO tumours (Figure 3d–g). The increase in vessel density and calibre was associated with reduced hypoxia in PAK4KO tumours (Figure 3i), indicating that the newly formed vessels may have a preserved sufficient perfusion capacity [57]. In contrast to PAK1KD, PAK4KO did not change the vascular normalisation markers (NG2/CD31 and α-SMA/CD31 ratios) or endothelial adhesion molecules (ICAM-1 and VCAM-1) in the absence of gemcitabine (Figure 4a–d). However, under gemcitabine treatment, PAK4KO exhibited a markedly increased ICAM-1 and VCAM-1 expression (Figure 4a,b), suggesting that a PAK4KO–gemcitabine combinational treatment may lead to stromal or vascular changes associated with ICAM-1 expression [58,59].

CD31 and CD34 are functionally distinct endothelial markers reflecting mature and immature vascular compartments [31,60]. In PAK1KD tumours, a marked reduction in CD31 with an unchanged CD34 expression suggests suppressed angiogenesis, which is consistent with reduced VEGFA levels. In contrast, PAK4KO tumours exhibited significant increases in the expression of both CD31 and CD34, indicating extensive endothelial proliferation and the coexistence of mature and immature vascular networks. These profiles implied that PAK1 inhibition led to vascular normalisation and a reduced vessel density, whereas PAK4KO promoted angiogenic expansion driven by fibronectin-associated signalling. From a therapeutic point, such vascular heterogeneity may influence gemcitabine distribution: normalised, but sparse, vessels in PAK1KD tumours might sustain the perfusion efficiency, whereas the dense, but immature, vasculature in PAK4KO tumours may increase the permeability, yet limit drug delivery. These findings highlight that the differential modulation of CD31^+^- and CD34^+^-defined vasculature by PAK1 or PAK4 could be strategically leveraged to optimise chemotherapy and vascular-targeted interventions in pancreatic cancer.

Furthermore, PAK4KO promoted VM formation (Figure 6a,b), an alternative vascularisation mechanism observed in aggressive PDA [61,62]. This upregulation of VM may further contribute to reduced hypoxia by providing additional perfusion routes independent of endothelial angiogenesis. However, VM structures in PAK4KO tumours exhibited a compromised integrity, as evidenced by a reduced VE-cadherin expression in tumour cells (Figure 6d), a critical component for maintaining the junctional stability in VM channels [63]. Conversely, endothelial-derived vessels showed an enhanced integrity (Figure 6c), suggesting a differential regulation of VE-cadherin between the tumour and stromal compartments. These findings indicate that PAK4 played a role in modulating VM, potentially through stemness or epithelial–mesenchymal transition pathways [61], and they highlight VM as a therapeutic target in PAK4-deficient PDA. The proteomic identification of upregulated EphA2 and EPH-ephrin pathway components in PAK4KO cells provides a mechanistic link, as EphA2 promotes VM in aggressive cancers by enabling tumour cell plasticity and vessel-like structure formation [64,65].

The proteomic study provided mechanistic insights into the vascular phenotypes observed in PAK-modified pancreatic cancer models. In PAK1KD cancer cells, the PPI network analysis highlighted significant changes in molecules involved in the VEGFA–VEGFR2 signalling pathway (reactome), which may underlie the reduced angiogenesis and enhanced vascular maturation observed in PAK1KD tumours (Figure 7b). The proteomic data also showed that the fibronectin expression increased by 2-fold in PAK4KO cancer cells (Figure 7c), indicating that the higher fibronectin deposition in PAK4KO tumours originated from cancer cells (Figure 3h). Moreover, the pathway enrichment analysis demonstrated that VEGF signalling pathway molecules (KEGG) were upregulated in PAK4KO cancer cells (Figure 7d). This molecular profile is consistent with the observed increase in angiogenesis and vessel calibre in PAK4KO tumours (Figure 3d–g), suggesting that the PAK4 knockout enhanced VEGF-associated signalling, despite a reduced VEGFA expression in these cells. Additionally, the upregulation of EphA2 and components of the EPH-ephrin signalling pathway in PAK4KO cells offered further insights into the mechanisms driving both angiogenesis and VM. EphA2, a member of the Eph receptor tyrosine kinase family, is overexpressed in various malignancies and promotes angiogenesis by regulating endothelial cell migration, adhesion, and vascular assembly through bidirectional signalling with ephrin ligands [66,67]. In pancreatic cancer, EphA2 contributes to tumour vascularisation by enhancing endothelial sprouting and tube formation [68]. Beyond traditional angiogenesis, EphA2 plays a pivotal role in VM, particularly in aggressive tumours such as glioma, ovarian, gastric, and prostate cancers, where it facilitates tumour cell plasticity, enabling cancer cells to adopt endothelial-like phenotypes and form perivascular vessel-like networks [40,64,69,70,71]. The broader EPH-ephrin signalling pathway mediates these effects through bidirectional communication, influencing cytoskeletal reorganisation, cell–cell interactions, and extracellular matrix remodelling—processes essential for both angiogenesis and VM [40,72]. In PAK4KO tumours, this pathway’s activation may further compensate for diminished VEGFA signalling, promoting alternative vascular strategies that sustain perfusion and contribute to the observed pro-angiogenic and VM phenotypes.

The upregulation of RhoA, ROCK1, and ROCK2 in PAK4KO cells further strengthens the molecular basis for enhanced VM. The RhoA/ROCK pathway is critical for cytoskeletal remodelling, cell motility, and contraction, which are essential for tumour cells to form tube-like structures in VM [46,73]. The inhibition of ROCK has been shown to suppress VM in various cancers, including hepatocellular carcinoma and melanoma, by disrupting actin cytoskeleton organisation and reducing cell migration [45,74]. In PAK4KO, the increased expression of RhoA/ROCK components may compensate for the loss of PAK4, which is typically involved in Rac/CDC42 signalling, by shifting towards RhoA-mediated pathways to drive VM formation [75]. This crosstalk between the PAK and RhoA/ROCK pathways highlights a potential adaptive mechanism in PAK4KO cells, promoting VM as an alternative to traditional angiogenesis.

An important consideration in interpreting our findings is that pancreatic cancer cells will acquire gemcitabine resistance through multiple mechanisms [76]. Among pancreatic cancer cell lines, PANC-1 has been reported to have the highest resistance to gemcitabine, with an IC50 of approximately 60 μM, which is about five-fold greater than that of MiaPaCa-2 cells [77]. MiaPaCa-2, another commonly used pancreatic cancer cell line, demonstrates a comparatively higher sensitivity to gemcitabine, making it a standard reference for assessing drug responsiveness. This resistance in PANC-1 is mediated, at least in part, through sustained ERK activity, as pharmacological MEK inhibition reduced ERK signalling and significantly enhanced gemcitabine sensitivity [78]. Similarly, combining gemcitabine with lenalidomide has been shown to overcome the resistance in PANC-1 cells by attenuating ERK activity [79]. GATA1 also enhances the gemcitabine resistance in PANC-1 cancer cells by activating antiapoptotic signalling, thereby reducing chemotherapy-induced cell death [80]. Furthermore, PANC-1 pancreatic cancer cells contain a side population with an inherently higher resistance to gemcitabine due to their increased expression of drug efflux transporters [19,81]. In our PANC-1-xenografted tumour model, gemcitabine alone reduced tumour growth, confirming its baseline anti-tumour activity, despite the intrinsic chemoresistance of this cell line. However, the efficacy of gemcitabine was not further enhanced by PAK1KD or PAK4KO, indicating that PAK modification did not overcome the higher resistance of the side population of PANC-1 cells to gemcitabine. Our previous report demonstrated that treatment with PF-3758309, a PAK4 inhibitor, produced maximal tumour growth inhibition when combined with gemcitabine in TKCC15-cell-derived tumours, which are comparatively more sensitive to gemcitabine (IC_50_ ≈ 5 nM) than PANC-1 cell lines [82]. In contrast, our recently published study using KPC-derived murine pancreatic cancer cells, which exhibit a high gemcitabine sensitivity, showed that PAK4KO enhanced the gemcitabine efficacy through an increased vessel diameter and the downregulation of DNA repair proteins, while PAK1KO reduced the chemotherapy response, despite suppressing tumour growth and promoting vascular normalisation [83]. This differential outcome highlights how the baseline chemosensitivity of the cell line—highly resistant human PANC-1 versus highly sensitive murine KPC—influences the interplay between PAK signalling modifications and gemcitabine responsiveness, suggesting that PAK targeting may be more effective in sensitising sensitive tumours to chemotherapy. Together, these findings indicate that the therapeutic efficacy of gemcitabine in the setting of PAK inhibition is limited by tumour-intrinsic resistance mechanisms, and that highly resistant tumours such as PANC-1 may necessitate a broader PAK blockade to achieve meaningful therapeutic benefits.

A limitation of this study is that functional assays of vascular perfusion, such as intravenous lectin injections, and a complementary hypoxia evaluation using pimonidazole staining were not performed. Instead, vascular normalisation was inferred from structural and molecular parameters, including the pericyte coverage (α-SMA/CD31 and NG2/CD31 ratios) and HIF-1α expression, which are widely accepted surrogates for vessel functionality [35,52]. Future studies integrating in vivo perfusion and pimonidazole assays will be valuable to validate the functional consequences of PAK1 or PAK4 modulation on vascular normalization and hypoxia in pancreatic cancer models.

Overall, our findings suggest that PAK1KD primarily reduced angiogenesis while promoting vascular normalisation, which is associated with enhanced pericyte coverage, reduced hypoxia, and a more permissive microenvironment for immune infiltration with ICAM-1 upregulation. In contrast, PAK4KO increased angiogenesis, which is associated with elevated fibronectin deposition and the enrichment of VEGF pathway molecules, while leading to stromal or vascular changes in the TME through the upregulation of endothelial adhesion molecules in combination with gemcitabine. Together, these results highlight the mechanisms through which PAK1 and PAK4 regulate the vasculature in pancreatic cancer, offering opportunities to exploit their differential functions for tailored therapeutic strategies.

A limitation of this study is that, although we employed human pancreatic cancer cells to better capture the clinical relevance of PAK modifications, the tumour microenvironment was derived from the murine host, which would have affected the stromal–vascular interactions and partially affected the interpretation of human-specific responses. In addition, the use of SCID mice precludes an evaluation of adaptive immune contributions. Future work in immune-competent and orthotopic humanised models will be critical to determine how PAK1- or PAK4-targeted interventions can affect the tumour vasculature, immune infiltration, and chemotherapy response in a clinically meaningful context.

## 5. Conclusions

This study identified distinct roles of PAK1KD and PAK4KO in modulating the tumour vasculature, angiogenesis, hypoxia, and tumour growth in pancreatic cancer models. PAK1KD inhibited tumour growth and was associated with reducing VEGFA-driven angiogenesis, promoting vascular normalisation and stromal or vascular changes associated with ICAM-1 expression, and reducing hypoxia. In contrast, PAK4KO suppressed tumour growth and was associated with increased angiogenesis and vessel diameter and reduced hypoxia in the TME. A combination of PAK4KO and gemcitabine led to stromal or vascular changes through the upregulation of the endothelial adhesion molecules ICAM-1 and VCAM-1. Together, these findings highlight the distinct mechanisms by which PAK1 and PAK4 regulate tumour growth, vasculature, and hypoxia.

## Data Availability

The data will be made available from the corresponding author upon request.

## Supplementary Materials

The following supporting information can be downloaded at: https://www.mdpi.com/article/10.3390/cells14221806/s1, Table S1: Buffers used in methods; Table S2: Primary antibodies for immunohistochemistry. N/A: not applicable; Table S3: Primary antibodies for immunofluorescence. N/A: not applicable; Table S4: Primary antibodies for Western blot. N/A: not applicable.

## Abbreviations

The following abbreviations are used in this manuscript:

PDA	Pancreatic ductal adenocarcinoma
TME	Tumour microenvironment
VEGFA	Vascular endothelial growth factor A
PAKs	P21-activated kinases
WT	Wild type
PAK1KD	PAK1 knockdown
PAK4KO	PAK4 knockout
NC	Negative control
sgRNAs	Single guide RNAs
FACS	Fluorescence-activated cell sorting
DMEM	Dulbecco’s modified Eagle’s medium
FBS	Foetal bovine serum
NGS	Normal goat serum
BSA	Bovine serum albumin
MVD	Microvessel density
H&E	Haematoxylin and eosin
RBCs	Red blood cells
CM	Conditioned media
LC	Liquid chromatography
DIA	Data-independent acquisition
MS	Mass spectrometry
LFQ	Label-free quantification
FDR	False discovery rate
PPI	Protein–protein interaction
SEM	Standard error of the mean
LSD	Least significant difference
VM	Vascular mimicry
PAS	Periodic acid–Schiff
EphA2	Ephrin type-A receptor 2
RhoA	Ras homolog family member A
ROCK	Rho-associated coiled-coil containing kinase
