# Supplementary material for: Targeting PAK1 or PAK4 Uncovers Different Mechanisms of Vascular Reprogramming in Pancreatic Cancer
Source: Cells. 2025 Nov 17;14(22):1806. doi: 10.3390/cells14221806 (PMC12650886; doi:10.3390/cells14221806)
Supplement: Supplementary file 1 [file cells-14-01806-s001.zip › cells-3920676-supplementary.pdf]

Supplementary Materials

Table S1. Buffers used in methods.

Buffers	Content
Tris-EDTA buffer	10 mM Tris base, 1 mM EDTA solution, 0.05% Tween 20, PH 9.0
TBS-T	20 mM Tris-HCl, 137 mM NaCl, 0.1% Tween 20, pH 7.6
2x loading buffer	125mM Tris, 20% glycerol, 4% SDS, 2.5% β-mercaptoethanol, Ph 6.8
Ripa buffer	25mM Tris HCL, 150mM NaCl, 1% Triton X-100, 1% Na deoxycholate, 0.5% SDS, 1mM EGTA, PH 8

Table S2. Primary antibodies for immunohistochemistry. N/A: not applicable.

Protein target	Dilution	Cat. number	Company	Clone name
CD31	1:1500	77699	Cell Signaling & Technology	D8V9E
CD34	1:15000	ab81289	Abcam	EP373Y
Fibronectin	1:16000	ab268020	Abcam	EPR23110-46
HIF-1α	1:500	ab179483	Abcam	EPR16897
ICAM-1	1:16000	ab179707	Abcam	N/A
VCAM-1	1:6000	ab134047	Abcam	EPR5047

Table S3. Primary antibodies for immunofluorescence. N/A: not applicable.

Protein target	Dilution	Cat. number	Company	Clone name
CD31	1:500-1000	77699	Cell Signaling & Technology	D8V9E
NG2	1:500	ab5320	Merck	N/A
α-SMA	1:5000	14395-1-AP	Proteintech	N/A
VE-cadherin	1:1000	ab33168	Abcam	N/A
Cytokeratin 19	1:6000	ab52625	Abcam	EP1580Y

Table S4. Primary antibodies for westernblot. N/A: not applicable.

Protein target	Dilution	Cat. number	Company	Clone name
ICAM-1	1:1000	67836	Cell Signaling & Technology	E3Q9N
VCAM-1	1:2000	ab134047	Abcam	EPR5047
VEGFA	1:2000	19003-1-AP	Proteintech	N/A
VE-cadherin	1:1000	ab33168	Abcam	N/A
PAK1	1:2000	2602	Cell Signaling & Technology	N/A

PAK4	1:2000	14685-1-AP	Proteintech	N/A
GAPDH	1:10000	2118	Cell Signaling & Technology	14C10
